# Refraining from spontaneous face touch is linked to personality traits, reduced memory performance and EEG changes

**DOI:** 10.1038/s41598-024-64723-z

**Published:** 2024-06-25

**Authors:** Kevin H. G. Butz, Stephanie M. Mueller, Jente L. Spille, Sven Martin, Martin Grunwald

**Affiliations:** Haptic Research Laboratory, Paul Flechsig Institute - Centre of Neuropathology and Brain Research, University of Leipzig, 04103 Leipzig, Germany

**Keywords:** Face-touch, Self-regulation, Memory, Behavioral inhibition, Self-infection, Human behaviour, Cognitive neuroscience

## Abstract

Spontaneous touches of one’s face (sFST) were suggested to serve cognitive-emotional regulation processes. During the pandemic, refraining from face-touching was recommended, yet, accompanying effects and the influence of personal attributes remain unclear. Ninety participants (45 female, 45 male) filled out a questionnaire concerning personality, anxiety screening and ADHD screening. Subsequently, they performed a delayed verbal memory recall task four times. After two times, sixty participants were instructed to refrain from face-touching (experimental group). Thirty participants did not receive behavioral instructions (control group). To identify face-touches and conduct further analysis, Video, EMG, and EEG data were recorded. Two samples were formed, depending on the adherence to completely refrain from face-touching (adherent, non-adherent sample) and compared to each other and the control group. EEG analyses uncovered that refraining from face-touching is accompanied by increased beta-power at sensorimotor sites and, exclusively in the non-adherent sample, at frontal sites. Decreased memory performance was found exclusively in subsamples, who non-adherently touched their face while retaining words. In terms of questionnaire results, lower Conscientiousness and higher ADHD screening scores were revealed by the non-adherent compared to the adherent sample. No differences were found among the subsamples. The presented results indicate that refraining from face-touching is related to personal attributes, accompanied by neurophysiological shifts and for a portion of humans by lower memory performance, supporting the notion that sFST serve processes beyond sensorimotor.

## Introduction

Spontaneous touches of one’s face (sFST) is a common behavior, independent of sociodemographic factors, such as age, gender or sexuality^1^. Approximately 40% of sFST, mostly without paying attention to the execution^1,2^, appears to be directed at the facial mucous membranes (eyes, nose, mouth) or proximal surrounding areas^3–8^. According to the World Health Organization (WHO) and the center for disease control and prevention (CDC), touching ones facial mucous membranes is a major transmission way to self-infect with the *Severe Acute Respiratory Syndrome—Coronavirus Type 2* (SARS-CoV2)^9,10^. Consequently, the interest in examining the occurrence and measures to reduce this behavior has increased recently [e.g. Refs.^4,11–17^]. One approach to reduce the occurrence of sFST and hence, the risk of self-infection, is the recommendation to refrain face-touching in public spaces. However, observational studies indicate a deficiency to voluntarily refrain from facial self-touches in a significant proportion of participants^2,11,18^, raising the question of the difficulty of refraining from face-touching.

An increased number of sFST has been observed in emotionally^19–25^ as well as in cognitively demanding situations^5,26^. Between the three seconds before and after sFST, significant neurophysiological changes have been found^26–28^. In contrast, few neurophysiological changes have been found for experimenter-requested, i.e. not spontaneous, facial self-touches^26,27^, supporting the assumption, that sFST serve purposes beyond sensorimotor functions. Assuming that sFST serve cognitive-emotional regulation processes raises the question of accompanying effects of refraining from face-touching.

**Figure 1:**
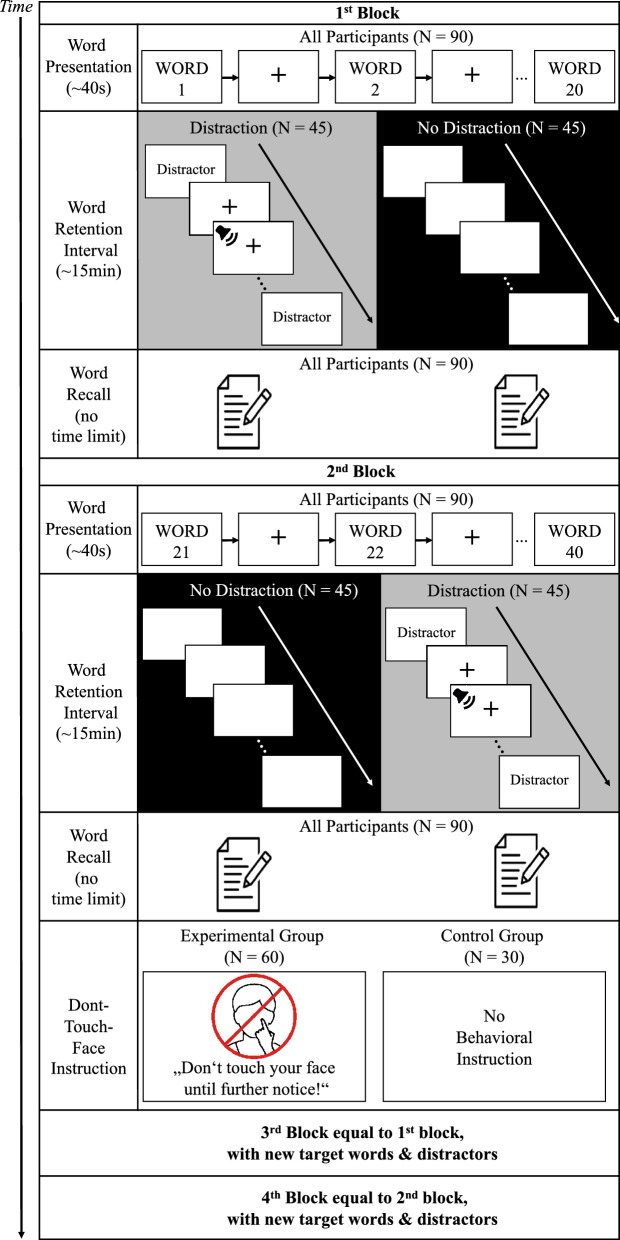
Experimental procedure. The experiment consisted of four similar blocks à three phases. In each block, ninety participants were first successively presented with twenty German nouns, which they were asked to retain in the following word retention interval. During the retention interval of each block, half of the participants were exposed to the distraction condition, the other half to the no-distraction condition. In the distraction condition, a fixation cross, auditory and semantic distractors were alternatingly presented in randomized order. In the no-distraction condition, a blank screen was presented. Subsequently, all participants were instructed to write down the words, which were to retain in this experimental block. Afterwards the next block started with new target words and distractors. Participants, who were presented with the distraction condition in block 1, were presented with the no distraction condition in block 2 and vice versa. Before the start of the third and fourth blocks, the experimental group received both semantic and graphical instructions not to touch their faces until further notice.

To the best of our knowledge, the only investigation of accompanying effects had revealed memory deterioration when refraining from face-touching in high frequently face-touching participants. This effect has not been observed for infrequently face-touching participants^29^. However, *Spille *et al. had immobilized the participants’ hands, i.e. actively prevented hand movements, which does not resemble voluntary refraining from face-touching as recommended during the pandemic. To address this gap, the study at hand investigated the capacity to and accompanying effects of voluntary refraining from facial self-touching by adapting a paradigm, tested in sFST studies^5,26–29^. Participants completed a delayed verbal memory recall task four times, each consisting of the three phases (a) word presentation/learning, successively presented on a screen (b) retention interval (RI) and (c) word reproductions. In each retention interval, participants were presented with either auditory and visually presented semantic distractors or a blank screen, which alternated between retention intervals for each participant. After the retention interval, participants wrote down the retained words. Before the third and fourth time of the memory task, two thirds of participants were instructed to refrain from facial self-touching, the remaining participants did not receive any instructions regarding face-touch behavior and served as control group (see Fig. 1). Throughout the entire experiment, EEG, EMG and video data were recorded.

In line with previous studies indicating a poor capacity in voluntary refraining from face-touching^2,11,18^, we anticipated that a significant number of participants in the experimental group would non-adherently execute sFST (Hyp. 1a), i.e. after being instructed to refrain from face-touching. As an increased number of sFST was observed in the presence compared to the absence of distractors^5,26^, we expected to observe more non-adherent face-touching throughout the experimental block with compared to without distraction (Hyp. 1b). Based on their face-touch behavior, for further analyses the experimental group was separated into two samples: (1) the non-adherent sample, who touched their face in any experimental phase after being instructed to refrain from face-touching, i.e. throughout the third and fourth experimental block and (2) the adherent sample, who did not touch their face after being instructed to refrain from face-touching.

After the performance of sFST, increased EEG power in the delta-, theta-, alpha-, beta- and gamma-band was reported and suggested that these increases reflect cognitive processes and, in the beta-band, motor processes^26,28^. Hypothesizing that the withdrawal of sFST is accompanied by a withdrawal of the reported power increase, we predicted decreased EEG power, except for the beta-band, after refraining from face-touching, more pronounced in the adherent sample (Hyp. 2a). As increased beta-band power was indicated to reflect motor inhibition at sensorimotor sites several times^30–32^, increased beta-power at these sites was expected to be more pronounced in the adherent sample (Hyp 2b). The right inferior frontal cortex (rIFG) has been indicated to play a pivotal role in neurophysiological changes after sFST^27^ and particularly, beta-band activity at this site during motor^33,34^ and cognitive inhibition^35^, hence, we predicted beta-band increase at the electrode covering the rIFG after refraining from face-touching, more pronounced in the adherent sample (Hyp. 2c).

Neurophysiological^26,28^ as well as observational studies^5,7^ have sFST suggested to be related to cognitive processes. Assuming that refraining from sFST interferes with this relation, we predicted to obtain lower memory performance in the adherent and non-adherent sample compared to the control group, after the instruction to refrain from face-touching (Hyp. 3a) As the adherent sample, per definition, completely refrained from face-touching, we predicted a lower memory performance for this sample compared to the non-adherent sample after the Don't-Touch-Face instruction (Hyp. 3b). Further, as more sFST have been observed in the presence of distractors than in the absence^5,26^ and distraction may interfere with verbal memory^36^, lower memory performance was predicted for the experimental block with than without distractors (Hyp. 3c).

As ADHD might influence capacity to refrain from face-touching^37^ as well as memory performance^38^, we examined ADHD differences and predicted increased ADHD scores in the non-adherent compared to the adherent sample (Hyp. 4a). As a positive relationship between anxiety and the number of sFST has been reported^20,25^, higher anxiety scores were expected in the non-adherent compared to the adherent sample (Hyp. 4b). Between personality traits and (facial) self-touches no relationship has been reported^29,39^. However, as higher conscientiousness, openness, and extraversion and lower neuroticism reported to be associated with better memory performance^40^, personality trait differences between the adherent and the non-adherent sample were expected (Hyp. 4c).

## Methods

### Participants

After providing written informed consent, ninety participants took part in the present experiment, forty-five who identified themselves as female, and forty-five as male. Inclusion criteria collected based on self-reporting were (1) age (20–40 years old); (2) native language (German); (3) no psychological or psychiatric conditions; (4) being naïve to EEG recordings and (5) normal or corrected-to-normal vision. Not being a German native speaker could have confounded the semantic memory processing and performance. Prior experience regarding EEG recordings might influence natural self-touch behavior in addition to the experimental situation. Inclusion criteria collected based on tests were (1) right-handedness (Edinburgh Inventory; Oldfield, 1971) and (2) no acute drug influence (Surestep Urine Test Drug Screen for 16 Drugs, Hangzhou AllTest Biotech Co., Ltd.). Participants were informed in advance by telephone that the drug test would be administered. A positive drug test for at least one of the sixteen tested substances led to the termination of the study appointment. In order to increase participants motivation in the memory task, participants were told to receive 40€ plus a bonus for good memory performance. All participants received a 45€ allowance for their participation (~ 18€/h). The study was approved by the Ethics Committee of University of Leipzig, Medical Faculty under the case number 426/20-ek. The procedures used in this study adhered to the tenets of the Declaration of Helsinki.

### Questionnaires

Before the EEG-experiment, participants filled out the following German questionnaires: (1) *NEO-FFI*^41,42^ (2) screening version of the *ADHD self-report scale* (ASRS)^43^ and (3) screening version of the *State and Trait Anxiety Inventory*^44,45^.

### Experimental procedure

Participants sat alone on an armchair in a room that served as a Faraday cage. Before the actual experiment began were block-randomly assigned to the experimental or the control group and it was tested, whether participants are able to hear the played sounds (audio device: TravelSound 200 (Creative, Germany)) and can read the words on the screen in front of them (screen: Emtec EMN22WI 22" (Emtec, Germany)). Experimental instructions, target and distractor words were presented on this screen, using a in-house script in Presentation^®^ software (Version 23.0, Neurobehavioral Systems, Inc., Berkeley, CA, www.neurobs.com). The experiment consisted of four similar blocks à three phases each in the order: 1. word presentation/learning (~ 40 s), 2. word retention interval (RI; ~ 15 min)) and 3. word reproduction (no time limit), for visualization see Fig. 1.

Prior to each word presentation/learning, participants were instructed to retain the words, which will be presented. After confirming to be ready, participants were successively presented with twenty German nouns, taken from the *Berlin Affective Word List – Revisited*^46^, balanced in arousal and valence ratings between experimental blocks (for details on the target words, please see Supplementary Table S1). Each word was presented for one second, between two words a fixation cross was presented for one second.

In the subsequent 15-min word retention interval, participants were presented with either the distraction or the no-distraction condition. In the distraction condition, the presentation of a fixation cross, auditory, or semantic distractors alternated. The order between the fixation cross, auditory and semantic distractors as well as the order for within each of the three was randomized. Auditory distractors were taken from the *International Affective Digitized Sounds 2* databank (IADS-2)^47^ and were balanced between experimental blocks with distraction in terms of arousal (for details on auditory distractors, please see Supplementary Table S3). Semantic distractors were taken from the *Berlin Affective Word List – Revisited*^46^ and balanced between experimental blocks and to the target words concerning arousal and valence ratings (for details on semantic distractors, please see Supplementary Table S2). Throughout each retention interval with distraction, 22 auditory distractors were altogether presented for ~ 265 s, 37 semantic distractors for ~ 185 s, and a fixation cross for ~ 455 s. During the no-distraction condition, participants were presented with a blank screen. Prior to the experiment, participants were randomly assigned to an experimental group or a control group. Forty-five participants started with the distraction condition in the first block, forty-five with the no-distraction condition. The distraction alternated blockwise for each participant, i.e. participants, who were presented with the distraction condition in the first block, were presented with the no distraction condition in the second block.

After the word retention interval, all participants were asked to recall the retained words from the word presentation/learning phase and write them down on a piece of paper with no time limit. Afterwards the next block began and twenty new words were presented, which were to retain. Prior to experimental blocks 3 and 4, sixty participants (experimental group) were instructed by text and a pictogram to refrain from touching their face until the end of the experiment (see Fig. 1). The remaining thirty participants (control group) did not receive any instructions regarding self-touch behavior.

### Data recording

EEG-, EMG- and video-data were recorded using the software Brain Vision Recorder software (v1.20.0801, Brain Products GmbH, Munich, Germany). EEG-Data from the following 19 Ag–AgCl electrodes/channels were recorded, positioned according to the International 10–20 system^48^: Fp1, Fp2, F7, F3, Fz, F4, F8, T3, C3, Cz, C4, T4, T5, T6, P3, Pz, P4, O1, O2 (online reference: linked earlobes). Eye movements were recorded using a vertical (VEOG) and a horizontal (HEOG) electrooculogram. Impedance was kept below 5 kΩ (EEG), and the sampling rate was set to 2000 Hz (EEG and EMG). EMG-electrodes were positioned on both arms (m. extensor carpi ulnaris), both legs (m. tibialis anterior), the right lower back (m. latissimimus dorsi) and the right neck (m. trapezius) of the participants. Video data were recorded with the camera Sony EVI-D70/D70P (Resolution: 720*576), which was invisible to the participants.

### Data pre-processing

All pre-processing steps were conducted in the software Brain Vision Analyzer (v2.2.1.8266, Brain Products GmbH, Munich, Germany). To determine spontaneous facial self-touch behavior, two independent raters (M.A. & C.T.S.) reviewed the data (EMG and Video) and marked the beginning and the end of each facial self-touch in the EEG/EMG data. Motor acts were considered as facial self-touch, when they met the following criteria (1) touching one’s face with right or left hand throughout the experiment (rest phases were excluded), (2) touching the vertical midline of the face, the ipsi- or contralateral side of the participants' face (touches to the hair, head, neck, or ears were excluded) and (3) no obvious functional reason for facial self-touch such as yawning, scratching or nose picking.

Pre-processing of the EEG-data involved the following steps: (1) filtering with an IIR filter (zero phase shift Butterworth filter, low cutoff 0.3 Hz, high cutoff 49 Hz, order 2, notch filter 50 Hz); (2) ocular artifact correction based on the vertical and horizontal ocular electrodes, using the algorithm of Ref.^49^; (3) automatic artifact correction (criteria: (a) Maximal allowed voltage step: 80 μV/ms—Mark as Bad: Before Event: 200 ms After Event: 200 ms; (b) Check difference (maxmin): Maximal allowed difference of values in intervals: 150 μV—Interval Length: 1000 ms—Mark as Bad: Before Event: 200 ms After Event: 200 ms and (c) Check Low Activity: Lowest allowed activity in intervals: 0.5 μV—Interval Length: 100 ms—Mark as Bad: Before Event: 200 ms After Event: 200 ms). If automatic artifact correction identified an artifact based on the above criteria, a segment starting 200 ms before and ending 200 ms after the marked artifact was excluded. Subsequently, the data for each retention interval and participant (four retention intervals per participant) were exported as one file each in the European Data Format (EDF +) format.

### Data analysis

All following data restructuring, computing and analyses were conducted in Matlab® (The MathWorks Inc., Version R2019 Update 4 (9.7.0.1296695)). First, EDF + retention interval files were successively imported into Matlab, using the function *edfread.m*. In a second step, the first and the last 60 s were extracted from each retention interval file (beginning and end period). In order to avoid biases by facial self-touches, EEG-data from 10 s before a facial self-touch to 10 s after a facial self-touch were excluded from further computing. The resulting data, beginning and end period, were each subdivided into 1 s-segments (see Supplementary Table S4 for details on included 1-s-segments). For each 1-s segment and each electrode, the mean power was calculated in 1-Hz-steps from 1 to 49 Hz, using the inbuilt *fft.m* function. A 10% Hanning window was applied. Subsequently, the power values were averaged over the 1-s segments, respectively for the beginning and the end period of each retention interval, resulting in one power-value for each participants, electrode, 1 Hz frequency step, period and retention interval (90 × 19 × 49 × 2 × 4). Afterwards, the data were averaged for the frequency bands delta (1–4 Hz), theta (4–8 Hz), alpha (8–12 Hz), beta (13–24 Hz), and gamma (24–49 Hz). Since more sFST were observed in the presence than in the absence of distractors^5,26^ and an increase in sFST was observed only when combining a memory task with distraction^26^, the EEG analyses were limited to the experimental blocks with distraction.

Based on their face-touch behavior, the experimental group (N = 60) was for further analyses separated into a non-adherent and adherent sample. A participant was assigned to the non-adherent sample if the following criterion was met: facial self-touching (as defined in the *Data pre-processing* section), in any experimental phase after the instruction to refrain from face-touching i.e. during the word presentation, retention interval or word reproduction of the third or fourth experimental block (please see Fig. 1). Vice versa, a participant was assigned to the adherent sample if they completely refrained from facial self-touching during the third and fourth experimental block.

### Statistics

One-way Anovas, paired and independent *t*-tests as well as Wilcoxon signed-rank or Wilcoxon ranksum tests were employed, as indicated by the respective test statistics (*F*, *t* or *z*), depending on the purpose and the normality of the respective distributions, as tested with the Shapiro–Wilk-test. Categorical variables were tested with the Chi-square test. Reported effects sizes are either Cohen's *d* for normally distributed data, the determinations-coefficient *η*^2^ = $\frac{{z}^{2}}{N}$^50^ for not normally distributed data, or partial eta squared for Anovas. For Cohen's d an effects size of *d* ≥ 0.2 is considered a small, *d* ≥ 0.5 a moderate and *d* ≥ 0.8 a strong effect. An effect size of *η*^2^ ≥ 0.02 is considered a weak effect, *η*^2^ ≥ 0.13 a moderate effect and η^2^ ≥ 0.26 a strong effect. To correct for multiple comparison, applied for neurophysiological data of each experimental block, the False-Discovery-Rate was controlled with the Benjamini–Hochberg procedure: 5 frequency-bands*19 electrodes = 95 tests^51^. Missing questionnaire data (0.3%) were replaced by the mean of existing data and did not affect statistical significance. Topoplots to visualize EEG-power differences were created with the Matlab-Toolbox BrainNet Viewer^52^ (http://www.nitrc.org/projects/bnv/).

### Ethics approval and consent to participate

The study was approved by the Ethics Committee of University of Leipzig, Medical Faculty under the case number 426/20-ek. The procedures used in this study adhered to the tenets of the Declaration of Helsinki. Informed consent was obtained from each participant before the study was conducted.

## Results

Ninety healthy right-handed participants performed a delayed verbal memory recall task four times, each consisting of (a) word presentation/learning, (b) retention interval and (c) word reproduction. During the retention interval, either the presentation of visual-semantic and auditory distractors alternated or a blank screen was presented. After two times performing the delayed verbal memory recall task, sixty participants (experimental group) were instructed to refrain from face-touching for the remaining experiment; the residual thirty participants did not receive any behavioral instruction (control group). Experimental and control group were gender-balanced. The experimental group did not significantly differ from the control group in terms of daytime of study (morning vs. afternoon; *χ*^2^ = 1.43, *p* = 0.23) or age (*z*_88_ = − 0.01,* p* = 0.99).

Throughout the entire experiment, 67 participants executed 438 sFST, of which 244 were executed during a retention interval. Significantly more sFST were executed during both retention intervals with distraction (M = 1.57, SD = 2.62, MD = 1 sFST) than during retention intervals without distraction (M = 1.14, SD = 2.19, MD = 0 sFST; *z*_89_ = 2.80, *p* = 0.005).

### “Don’t-Touch-Face” – a significant proportion of participants still touched their face

As predicted, among the sixty participants of the experimental group, instructed to refrain from face-touching, thirty individuals touched their face a total of 82 times after the instruction (referred to as the *non-adherent sample* from here). The thirty participants, who did not touch their face after being instructed to refrain from face-touching, are referred to from here as the *adherent sample*. No significant differences between the adherent and the non-adherent sample were obtained in terms of age (adherent: M = 26.7, SD = 4.86, MD = 26 years; non-adherent: M = 25.93, SD = 4.62, MD = 25 years ; *z*_*58*_ = 0.549, *p* = 0.583); group membership (*χ*^2^ = 0.267, *p* = 0.606); daytime of study (morning vs. afternoon; *χ*^2^ = 0, *p* = 1) and gender (*χ*^2^ = 0.267, *p* = 0.606). In the experimental group significantly fewer face touches were observed after the instruction to refrain from facial self-touching (block 3&4: M = 1.37, SD = 2.32, MD = 0.50 sFST) than before this instruction (block 1&2: M = 2.85, SD = 4.87, MD = 1 sFST; *Z*_59_ = 2.81, *p* = 0.0049, *η*^2^ = 0.13). In contrast, the control group, which was not instructed to refrain from facial self-touching, executed an increased number of face-touches during block 3 and 4 (M = 3.47, SD = 5.26, MD = 2.50 sFST) compared to block 1 and 2 (M = 1.47, SD = 2.43, MD = 1.00 sFST), at the border to statistical significance (*Z*_29_ =  − 1.908, *p* = 0.056, *η*^*2*^ = 0.12).

In the first two experimental blocks, i.e. before the experimental group was instructed to refrain from face-touching, the non-adherent sample performed more sFST (M = 4.73, SD = 6.23, MD = 2 sFST) than the adherent sample (M = 0.97 SD = 1.50 MD = 0 sFST; *Z*_58_ = 2.80, *p* = 0.005, η^2^ = 0.26). The control group performed less sFST (M = 2.60, SD = 3.01, MD = 2 sFST), yet not significant, than the non-adherent sample (*Z*_58_ = 1.29, *p* = 0.20), but significantly more than the adherent sample (*Z*_58_ = − 2.75, *p* = 0.006).

### Refraining from face-touching affects the adherent and non-adherent sample neurophysiological differently

**Figure 2:**
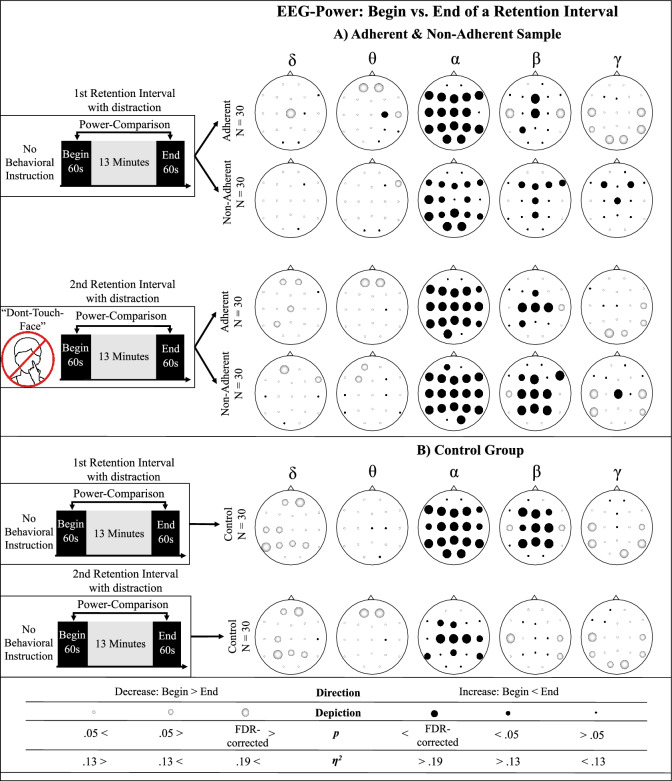
Intragroup comparisons of EEG-Power changes while (not) refraining from face touching. Results of EEG-power comparisons between the first and the last sixty seconds of retention intervals with distraction for the frequency bands delta, theta, alpha, beta and gamma, separated into the (**A**) experimental group (non-adherent adherent sample) and (**B**) control group. All comparisons were conducted with repeated measure Wilcoxon signed rank tests. Prior to the second retention interval with distraction ((**A**) bottom row), the experimental group was instructed to refrain from face-touching, the control group did not receive any behavioral instruction. Non-Adherent sample = Participants, who touched their face, in any experimental phase after being instructed to refrain from face-touching. *FDR* false discovery rate, correction for multiple comparisons^51^.

To investigate whether refraining from face-touching manifests neurophysiologically, intragroup comparisons between the first and the last sixty seconds of retention intervals with distractors were compared separately for the control group, the adherent and the non-adherent sample. (see Fig. 2A and B).

To determine neurophysiological shifts, not related to refraining from face-touching, EEG power was compared between the first and the last sixty seconds of the first experimental block with distractors, i.e. before the experimental group was instructed to refrain from face-touching (see the upper row of Fig. 2A and B). In all group/samples, significant alpha-power increases were measured at multiple electrodes, yet, less widespread in the non-adherent sample. For the adherent sample, a significant beta-power decrease at bilateral temporal sites was measured, which was also found for the control group as a statistical trend, but not for the non-adherent sample. Increased fronto- and centro-central beta-power was exclusively measured for the adherent sample and the control group. Additionally, increased beta power was measured at parietal electrodes for the control group. Decreased temporal and occipital gamma-power was obtained exclusively for the control group and the adherent sample (for detailed statistical results, see Supplementary Tables S7, S8 and S9).

To examine neurophysiological shifts related to the refraining from facial self-touching, EEG power was compared between the first and the last sixty seconds of the second retention interval with distraction, i.e. after the experimental group was instructed to refrain from facial self-touches (see the bottom row of Fig. 2A and B). Increased alpha-power was measured at sensorimotor sites in the control group and at nearly all sites in the adherent and the non-adherent sample. While increased beta-band power was measured at sensorimotor sites for the adherent sample and at fronto-central, right fronto-lateral, sensorimotor and parietal sites for non-adherent participants, no beta-power increase was measured for the control group. Gamma-band power increase at the centro-central site was exclusively measured for the non-adherent sample (for detailed statistical results, see Supplementary Tables S7, S8 and S9).

### Lower memory performance in non-adherent subsample, but not the non-adherent sample

To test whether the instruction to refrain from face-touching is accompanied by memory performance changes, we performed a repeated measures Anova, which revealed a significant main effect for the factor condition (before and after the Dont-Touch-Face instruction) [*F*(1, 88) = 4.22, *p* = 0.04, η^2^_p_ = 0.46], a trend for the factor group (control group, experimental group) [*F*(1, 88) = 2.79, *p* = 0.098 η^2^_p_ = 0.03] and no significant interaction between condition and group [*F*(1, 88) = 0.41, *p* = 0.48, η^2^_p_ = 0.01]. Post-hoc tests revealed a statistical trend for a memory performance increase in the control group (*t* = − 1.69, *p* = 0.094) and no significant differences for the experimental group between before and after the instruction to refrain from face-touching (*t* = − 0.58, *p* = 0.25).

Separating the experimental group into the adherent sample, that completely refrained from face-touching after the Dont-Touch-Face instruction, and the non-adherent sample, that touched their face in any experimental phase after the Dont-Touch-Face instruction, and comparing the memory performance between the two samples revealed no significant main effect for the factor condition [*F*(1, 58) = 1.43, *p* = 0.24, η^2^_p_ = 0.02] and the factor group [*F*(1, 58) = 0.02, *p* = 0.89, η^2^_p_ = 0.00].

To investigate whether non-adherent face-touching in temporal proximity to a memory recall is accompanied by lower memory performance, the samples were adjusted for an exploratory analysis, resulting in subsamples, which were separated by distraction conditions. Instead of considering all participants as non-adherent, who touched their face in any experimental phase after the “Don’t-Touch- Face”-instruction, only those were considered in a non-adherent subsample if non-adherent face-touching was executed throughout a retention interval prior to the respective memory recall. The adherent subsample consisted of participants who did not touch their face in the retention interval of an experimental block after the “Don’t-Touch-Face” instruction. The adjusted subsamples consisted for the condition with distractors of eleven non-adherent and forty-nine adherent participants, for the condition without distractors of eight non-adherent and fifty-two adherent participants. The subsamples did not significantly differ in terms of gender, age, group membership and daytime of study (see Supplementary Table S5).

A repeated measure Anova with the factors condition (first and second block with distraction) and group (control group, non-adherent and adherent subsample) revealed no significant main effect for condition [*F*(1, 87) = 0.93, *p* = 0.34, η^2^_p_ = 0.01], no significant interaction between condition and group [*F*(2, 87) = 1.66, *p* = 0.20, η^2^_p_ = 0.04] and a significant group effect [*F*(2, 87) = 0.3.34, *p* = 0.04, η^2^_p_ = 0.07]. Post-hoc tests for the second experimental block with distractors, i.e. after the experimental group was instructed to refrain from face-touching, revealed significantly more correctly recalled words for the control group (M: 10.27, SD: 3.63, MD: 9 words; *t*_39_ = 3.08, *p* = 0.003) and the adherent subsample (M: 9.45, SD: 2.80, MD: 10 words; *t*_58_ = 2.49, *p* = 0.015) compared to the non-adherent subsample (M: 6.82, SD: 3.43, MD: 7 words).

**Figure 3:**
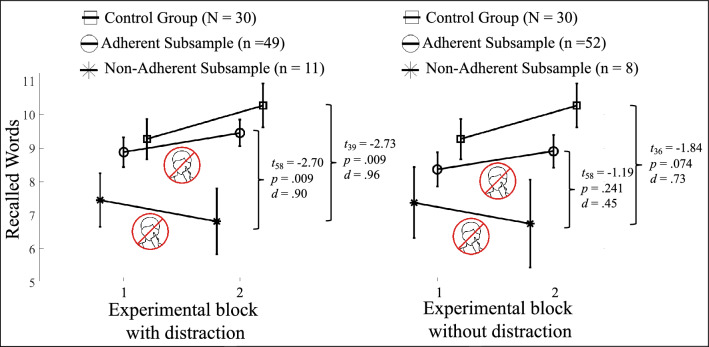
Line plot of mean correctly recalled words. Correctly recalled words of the first and the second memory task with and without distraction, respectively, separated into control group and experimental group (adherent and non-adherent subsamples). The experimental group was instructed to refrain from face-touching prior to the respective second block. Comparisons were conducted with independent t-tests. Adherent subsample = Participants, who did not touch their face during the retention interval of the second run (distraction – n = 49, no distraction – n = 52). Non-adherent subsample = Participants, who touched their face at least once throughout a retention interval after being instructed to refrain from face touching (distraction – n = 11, no distraction – n = 8). Error bars indicate the standard error. *d* = Cohen’s *d* (effect size).

The respective Anova for the condition without distractors did not reveal a significant main effect for condition [*F*(1, 87) = 0.02, *p* = 0.89, η^2^_p_ = 0.00], no significant interaction between condition and group [*F*(2, 87) = 0.75, *p* = 0.48, η^2^_p_ = 0.02] and no significant group effect [*F*(2, 87) = 1.78, *p* = 0.18, η^2^_p_ = 0.04]. Post-hoc tests for the second experimental block without distractors revealed a statistical trend for more correctly recalled words for the control group (M: 10.40, SD: 3.36, MD: 11 words; *t*_39_ = 1.74, *p* < 0.01) and but not the adherent subsample (M: 9.44 SD: 3.66, MD: 10 words; *t*_58_ = 1.15, *p* = 0.26) compared to the non-adherent subsample (M: 7.88, SD: 4.12 MD: 8 words). Mean recalled words for each run and group/subsample as well as groupwise comparisons are visualized in Fig. 3.

Correlational analyses involving the memory performance and number of face-touches, performed during the retention interval prior to the memory recall of an experimental block, revealed a significant negative relationship between the non-adherent sample and the memory performance in the second retention interval with distractors. Correlational results for all participants as well as separated into control group, adherent and non-adherent subsamples can be found in Table 1.

**﻿Table 1:** Correlation results involving the number of sFST, performed throughout a retention interval (RI), and correctly recalled words, separated by group/sample and RI.

Rho (*p*)	Correctly recalled words			
sFST	1st RI with distractors	1st RI without distractors	2nd RI with distractors	2nd RI without distractors
All participants (N = 90)	− 0.21* (0.045)	0.02 (0.87)	− 0.24* (0.02)	− 0.04 (0.73)
Control group (N = 30)	− 0.17 (0.37)	− 0.20 (0.28)	− 0.30 (0.10)	− 0.07 (0.73)
Adherent subsample with/without distractors (n = 49/52)	− 0.20 (0.17)	− 0.16 (0.26)	0^&^	0^&^
Non-adherent subsample with/without distractors (n = 11/8)	0.12 (0.73)	0.05 (0.91)	− 0.19 (0.57)	− 0.19 (0.65)

Non-adherent sample = at least one face-touch throughout any experimental phase, after being instructed to refrain from face-touching, i.e. throughout the third and fourth experimental block. Non-adherent subsample = at least one face-touch throughout the retention interval (RI) of an experimental block, after being instructed to refrain from face-touching, i.e. throughout the RI of the third or fourth experimental block. *p < 0.05.

^&^No data for the second retention interval of both, with and without distractors as the subsample did, per definition, not perform any sFST in the second retention intervals.

### Non-adherence to Dont-Touch-Face instruction associated with higher ADHD screening and conscientiousness scores

Prior to the memory task, participants filled out the questionnaires *NEO-Five Factor Inventory*^41,42^, to measure the BIG FIVE personality dimensions (Neuroticism, Extraversion, Openness, Agreeableness and Conscientiousness), the screening version of the *ADHD self-report scale*^43^ and the screening version of the *State and Trait Anxiety Inventory*^44,45^.

**Figure 4﻿:**
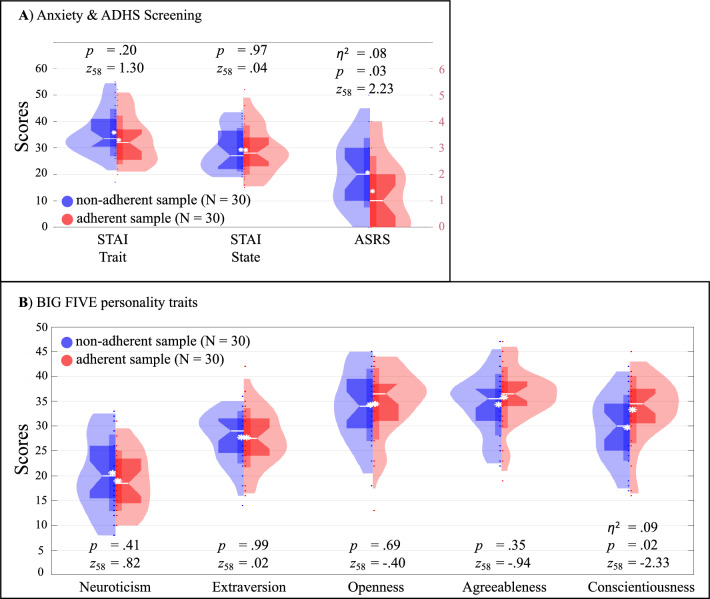
Questionnaire results. Questionnaire results and statistical comparisons between the non-adherent and the adherent sample as violin plots, as compared with Wilcoxon ranksum tests. (**A**) State and trait anxiety inventory (STAI; range 0–60) and Adult ADHD self-report scale results (ASRS; range 0–6). (**B**) BIG FIVE personality dimensions as measured by the NEO-FFI. Non-adherent sample = Participants, who touched their face, after being instructed to refrain from face-touching. White stars indicate the mean, white lines the median. *η*^2^ = Determination coefficient (effect size).

In terms of the BIG FIVE personality traits, significantly lower *Conscientiousness* values were obtained for the non-adherent compared to the adherent sample (*Z*_*58*_ = − 2.33, p = 0.02, η^2^ = 0.09). No significant differences were found for the remaining four BIG FIVE personality measures (see Fig. 4B). In the Adult ADHD Self-Report Scale (ASRS; screening), significantly higher values were found for the non-adherent compared to the adherent sample (*Z*_*58*_ = 2.23,* p* = 0.03, η^2^ = 0.08). Neither for state (*Z*_*58*_ = 0.04, *p* = 0.97) nor for trait anxiety (*Z*_*58*_ = 1.30, *p* = 0.20) significant differences were found between the adherent and the non-adherent sample (see Fig. 4A). No significant difference was obtained in any questionnaire dimension between the experimental group (adherent and non-adherent sample) and the control group (for details see Supplementary Table S6).

To test whether the differences in questionnaire results still can be found after the adjustment of the criterion, we re-analyzed the data, comparing the control group, the adherent and non-adherent subsamples instead of the initially formed adherent and non-adherent sample. No significant differences were found in terms of ADHD screening, anxiety screening and personality (Table 2).

**Table 2:** Results of one-way anovas comparing of the questionnaire results between the control group and the subsamples.

	Factor group (control group N = 30, adherent n = 49, non-adherent n = 11, subsample with distractors)		Factor group (control group N = 30, adherent n = 52, non-adherent n = 8, subsample without distractors)	
	F(2, 87)	*p*	F(2, 87)	*p*
STAI—trait	0.92	0.40	0.27	0.76
STAI—state	0.76	0.47	10.19	0.33
ASRS	0.41	0.67	0.49	0.62
Neuroticism	0.30	0.74	0.29	0.75
Extraversion	0.84	0.44	0.72	0.50
Openness	1.30	0.28	0.31	0.74
Agreeableness	0.53	0.60	0.51	0.61
Conscientiousness	0.40	0.68	0.49	0.62

Non-adherent subsample = at least one face-touch throughout the retention interval (RI) of an experimental block, after being instructed to refrain from face-touching, i.e. throughout the RI of the third or fourth experimental block.

*STAI* state and trait anxiety inventory screening, *ASRS* ADHD screening; (neuroticism, extraversion, openness, agreeableness and conscientiousness) – Big five personality dimensions as measured with the NEO-FFI.

## Discussion

It was suggested that spontaneous facial self-touches (sFST) can influence processes beyond sensorimotor ones^5,26–29^. Throughout the SARS-CoV2 pandemic, we were recommended to refrain from face-touching. To examine the capacity to and accompanying effects of voluntarily refraining from sFST, we recorded face-touch behavior, memory performance and neurophysiological data of ninety participants, throughout four times of a delayed verbal memory recall task. Prior to the third and fourth experimental block, sixty participants were instructed to refrain from face-touching.

In line with Hyp. 1a and previous findings^2,11,18^, half of the participants engaged in face-touching despite being instructed not to (non-adherent sample), potentially rendering an individual risk, as face-touching was claimed to be one of three major ways to self-infect with SARS-CoV2. The reduced number of face-touches after the Dont-Touch-Face instruction might indicate public recommendations to refrain from face-touching being effective in reducing the occurrence of this behavior. In accordance with a previous study and Hyp. 1b, that reported an increased number of sFST only when combining a memory task with distractor presentation, but not without^26^, more sFST were performed during the retention intervals with compared to without distractors. *Grunwald *et al. speculate that distractor interfere with the maintenance of task-related memory content and that neurophysiological changes following the performance of sFST reflect “processes of working memory maintenance as well as down regulation of negative emotions during sensorimotor integration processes”^26^.

EEG power analyses after (not) refraining from face-touching while retaining words and being presented with distractors, revealed several different changes. However, opposing our prediction (Hyp. 2a), no power changes in the frequency bands delta and theta were found, which might be due to employing short-term neurophysiological effects in the course sFST^26,28^ to predict long-term effects of refraining from face-touching.

In the alpha-band, a widespread power increase in the alpha-band was found (Hyp. 2a). As this effect was found in all groups and both the experimental run before and after participants were instructed to refrain from face-touching, these findings likely reflect the inhibition of task-irrelevant stimuli, such as distractors^53–55^. Less widespread alpha power increase in the first retention interval, as found in the non-adherent sample, may reflect deviating capacities to inhibit task-irrelevant stimuli. In the second retention interval with distraction, less widespread alpha power increase was found in the control group. As they may have habituated to the distraction and were not additionally strained by behavioral instructions, these findings may reflect lower inhibitory effort.

Beta-power increase was, as predicted (Hyp. 2b), found at central sites in the adherent and non-adherent sample, but not the control group, after the instruction to refrain from face-touching, which might reflect long-term effects of motor inhibition^30–32,56^. Contrasting our prediction, the beta-power increase was more widespread in the non-adherent sample, involving fronto-central and parietal sites, which may imply the engagement of the parieto-frontal network and increased efforts in motor planning and updating in the non-adherent sample^57^.

In line with our expectation (Hyp. 2c), increased beta power was spread to the right inferior frontal gyrus (rIFG) in the non-adherent, yet not the adherent sample. The rIFG was suggested to play a pivotal role in motor inhibition^34,58–60^ as well as in the course of sFST^27^. As the non-adherent sample overall revealed a higher number, refraining from face-touching might require increased inhibitory effort. As the rIFG was argued to be part of a broader inhibitory network, involving cognitive inhibition^35^ as well as regulation of emotions^61–63^, the results at hand might reflect increased inhibitory efforts in the non-adherent sample beyond the motor system. To enlighten the relationship between spontaneous facial self-touches and activity in the right frontal inferior cortex, future studies could examine whether accompanying effects of refraining from facial self-touches are directly related to activity at this site.

In the gamma-band, the expected power decrease was found at posterior sites in the adherent and, more pronounced, in the non-adherent sample after they were instructed to refrain from face-touching (Hyp. 2a). While a less widespread gamma-band decrease was found in the adherent sample after the Don’t-Face-Touch instruction, a more widespread gamma-band decrease was found in the non-adherent sample. As a positive relationship between gamma-band power and memory performance has been reported^64^, the gamma-power decrease in the non-adherent sample may indicate at least momentary decreased cognitive capacities.

In contrast to the expected lower memory performance in the experimental group compared to the control group after the former were instructed to refrain from face-touching, no memory performance differences were revealed, neither between the control group and the entire experimental group (Hyp. 3a) nor after separate the experimental group into the adherent and non-adherent sample (Hyp. 3b), neither for the condition with nor without distractors (Hyp. 3c). However, the revealed negative relation between the number of sFST, performed during a retention interval with distractors, and the respective memory performance might indicate that the initial criterion to form the adherent and non-adherent sample did not reflect the temporal specificity of sFST, which are assumed to be “instantaneous responses to sudden attentional distractors”^5^.

To test whether refraining from face-touching while retaining words, i.e. during the retention interval (RI) of an experimental block, is accompanied by lower memory performance, we re-analyzed the data and compared participants who non-adherently face-touched during the retention interval of an experimental block (non-adherent subsamples) to those who did not (adherent subsamples). No memory differences were found between the groups before the instruction to refrain from face-touching. However, after the second RI with distraction, i.e. after being instructed to refrain from face-touching, the control group as well as the adherent subsample showed significantly better memory performance than the non-adherent subsample. The worse memory performance, found in a non-adherent subsample, is in line with earlier findings that showed forced refraining from face-touching to be accompanied by memory performance deterioration^29^. Lower memory performance, exclusively obtained from the subsample that non-adherently touched their face during the retention interval with distraction, in contrast to non-adherent face-touching in all experimental phases, suggest face-touching to reflect a momentary state affecting memory. The memory performance of the adherent subsamples, which resembled the performance of the control group might on the one hand suggest learning effects and on the other hand that the adherent subsamples were not substantially affected by the behavioral task to refrain from face-touching.

Contrary to *Spille and colleagues*^29^, the task to voluntarily refrain from face-touching might be particularly challenging to adhere to for participants who frequently touch their face, which could increase the cognitive load and affect memory performance. However, no relation was found between the number of non-adherent sFST, performed during an RI, and memory performance. Further, increased face-touching and slightly lower memory performance obtained from the non-adherent subsamples even before the instruction to refrain from face-touching might suggest an underlying factor, influencing both memory performance and face-touch behavior. Examining ADHD, anxiety or personality traits as confounding factors of memory performance^38^ or the capacity to refrain from face-touching^20,25,36,37^, revealed no differences between the control group and the subsamples, indicating that the revealed memory performance differences are not due to differences in these dimensions.

The initially formed non-adherent sample revealed lower *Conscientiousness* scores compared to the adherent sample, that completely refrained from face-touching following the Dont-Touch-Face instruction (Hyp. 4c). *Conscientiousness* was described as “control [of] one’s behavior in service of one’s goals. Individuals with low conscientiousness have difficulty sticking to a schedule, are disorganized, and can be unreliable”^65^. As a substantial body of literature indicating *Conscientiousness* as a predictor for traits, such as self-regulation^66^, and academic success^67^, face-touch behavior might also be, in light of the results, a valuable predictor. To clarify the predictive value and whether the relation between *Conscientiousness* and self-touch is limited to the face, future studies are needed.

In line with our prediction (Hyp. 4a) and literature reporting a negative relationship between measures of *Conscientiousness* and ADHD^68^, higher ADHD screening scores were obtained from the non-adherent compared to the adherent sample. As the number of face-touches in the non-adherent compared to the adherent sample was increased already before the experimental group was instructed to refrain from face-touching, the results at hand might reflect ADHD related hyperactivity. In ADHD, a reduced capacity to inhibit different behaviors in ADHD was reported several times^37^, querying whether the incapacity to refrain from behavior is limited to face-touches. Self-touch has been reported to be associated with the attenuation of stress-related cortisol level increase, yet, not differing between self-touch targets (body, face)^69^. As ADHD was measured with a screening questionnaire in the present study, the results should be interpreted cautiously. To clarify, whether ADHD diagnosis is accompanied by an increased face-touch frequency and the specificity of the face as a self-touch target, future studies could compare self-touches of the body and the face between humans with and without ADHD diagnosis.

Contrasting our hypothesis (Hyp. 4b) and previous reports^20,25^, no differences were obtained in terms of state and trait anxiety measures between the adherent and non-adherent sample nor the control group, indicating that anxiety does not determine the capacity to refrain from face-touching. To further enlighten the relationship between anxiety and (facial) self-touch behavior, more studies are needed.

### Limitation

In the study at hand, the research object was the capacity to and accompanying effects of refraining from facial self-touches, as recommended under natural circumstances (e.g. in public transportation) throughout the pandemic. While refraining from facial self-touching in everyday situations might be associated with the reward of potentially reducing the risk for self-infection, no reward was provided for successfully refraining from face-touching. Hence, the generalizability of our results under laboratory circumstances to real-world settings may be limited.

Besides cognitive processes, spontaneous facial self-touches were suggested to serve emotional regulative mechanisms^5,26,28^, both assumed to be intertwined^70^. While anxiety state measures did not reveal differences between the examined group/(sub)samples, other emotional states were not examined, which might confound the present results.

## Future direction & conclusion

The likelihood of experiencing a pandemic in a lifetime has been reported to rise^71^, which might increase the risk of infection transmission through touches of facial mucous membranes. Considering the results at hand in terms of capacity and accompanying effects, voluntarily refraining from face-touching seems to be challenging and to bear consequences for at least a proportion of humans. To reduce manual face-touching behavior and avoid unwanted consequences of refraining from face-touching, future studies could explore the feasibility of replacing manual face-touches by alternative behaviors, such as face-touching with an object made out of antibacterial material or other types of self-touch behavior, not targeting mucous membranes.

To conclude, the presented study examined the capacity to and accompanying effects of refraining from face-touching. Half of the participants engaged at least once in face-touching after being instructed not to and displayed overall higher face-touch frequency, higher ADHD screening scores, lower *Conscientiousness* scores and neurophysiological differences. A small portion of participants, that nevertheless touched their face while retaining words under additional cognitive-emotional strain, revealed lower memory performance after being instructed to refrain from face-touching. The study suggests that sFST influence processes beyond sensorimotoric, yet, requiring further research.

### Supplementary Information

Supplementary Tables.

## Data Availability

The datasets used and/or analysed during the current study are available from the corresponding author on reasonable request.

## Abbreviations

ADHD: Attention deficit hyperactivity disorder
ASRS: Adult ADHD self-report scale
FDR: False discovery rate
rIFG: Right inferior frontal gyrus
SARS-CoV2: Severe acute respiratory syndrome – coronavirus type 2
sFST: Spontaneous facial self-touch
STAI: State and trait anxiety inventory
RI: Retention interval
