# Supplementary material for: Refraining from spontaneous face touch is linked to personality traits, reduced memory performance and EEG changes
Source: Sci Rep. 2024 Jun 25;14:14600. doi: 10.1038/s41598-024-64723-z (PMC11199707; doi:10.1038/s41598-024-64723-z)
Supplement: Supplementary file 1 — Supplementary Tables. [file 41598_2024_64723_MOESM1_ESM.pdf]

Refraining from Spontaneous Face Touch is Linked to Personality Traits, Reduced Memory Performance and EEG Changes

Kevin H.G. Butz[1], Stephanie M. Mueller[1], Jente L. Spille[1], Sven Martin[1], Martin Grunwald[1]*

[1]University of Leipzig, Paul Flechsig Institute, Centre of Neuropathology and Brain Research, Haptic Research Laboratory, 04103 Leipzig, Germany

*Corresponding Author: mgrun@medizin.uni-leipzig.de

Supplementary material

Tab. S1 – Target words presented in the semantic memory task

Target Word Block 1	EMO_ MEAN	AROUSAL_ MEAN	Target Word Block 2	EMO_ MEAN	AROUSAL_ MEAN
Abbild	-0.20	2.11	Archiv	0.45	1.89
Anbau	0.85	1.58	Ausbau	0.62	1.63
Balken	0.30	2.11	Besteck	0.59	2.00
Becher	0.55	1.71	Betrieb	-0.24	2.11
Distel	-0.55	2.16	Hose	0.77	2.00
Faden	0.20	1.80	Kloster	0.06	1.83
Japan	0.25	2.11	Kupfer	0.50	1.94
Kiesel	0.50	1.61	Leim	0.00	2.11
Leinwand	0.40	1.94	Luftraum	0.60	2.11
Mitte	0.75	2.11	Meter	0.15	2.16
Pinsel	0.55	1.97	November	-0.50	2.17
Regal	0.55	2.05	Pappe	-0.05	1.94
Schuh	1.00	2.05	Paste	-0.15	1.94
Seil	0.18	2.18	Quote	-0.05	2.06
Sohle	0.00	1.78	Raupe	0.30	1.84
Stufe	0.30	2.15	Runde	0.30	1.84
Tablett	0.23	1.91	Seite	0.25	1.89
Tag	0.97	2.10	Teller	0.59	1.59
Toaster	0.82	1.68	Unkraut	-1.00	1.86
**Target Word Block 3**	**EMO_ MEAN**	**AROUSAL_ MEAN**	**Target Word Block 4**	**EMO_ MEAN**	**AROUSAL_ MEAN**
Ablauf	0.00	2.11	Anzahl	0.20	1.71
Besen	-0.25	2.05	Bereich	0.03	1.78
Brot	0.91	1.95	Fächer	0.68	2.14
Dokument	0.60	2.10	Kegel	0.35	2.00
Finger	0.70	2.11	Kugel	0.45	2.00
Gegend	0.30	1.94	Lampe	0.85	2.14

Hafer	0.75	1.47	Lesung	0.40	2.00
Henne	0.25	2.06	Monat	0.30	2.11
Kanzel	-0.85	1.95	Nichte	0.45	2.00
Kiste	0.45	1.89	Ordner	-0.23	2.05
Laken	0.45	1.89	Paprika	1.00	1.91
Löffel	0.59	2.14	Person	0.50	2.06
Moorbad	0.20	1.94	Pflege	0.45	2.00
Platz	0.38	2.12	Quader	0.10	1.88
Regen	0.32	2.06	Schrank	0.27	1.77
Skat	0.00	2.00	Teppich	0.36	1.82
Sorte	0.40	2.00	Titel	0.65	1.89
Verfügung	-0.38	1.95	Vormund	-0.60	2.16
Walnuss	0.86	1.86	Wolke	1.00	1.89

Emotional Valence (EMO) and Arousal ratings for target words words as reported in Võ et al. (2009) for the four experimental blocks.

Tab. S2 – Semantic distractors presented during the retention intervals

Distraction Word Block 1/2	EMO_ MEAN	AROUSAL_ MEAN	Distraction Word Block 3/4	EMO_ MEAN	AROUSAL_ MEAN
Abteil	-0.15	2.12	Achse	0.00	2.17
Anker	1.00	1.89	Basis	0.35	1.72
Bach	1.00	1.78	Blei	-0.62	1.89
Birne	1.00	1.72	Bohne	0.65	1.58
Brunnen	0.55	1.86	Cello	0.95	2.09
Deckel	-0.09	1.86	Daumen	0.45	1.75
Eindruck	0.40	2.18	Dienst	-0.76	2.14
Flasche	0.45	1.95	Eule	0.64	2.09
Glas	0.64	1.77	Ferse	0.20	2.13
Hemd	0.68	1.95	Finder	0.75	1.89
Hering	-0.05	2.16	Fliege	-0.95	2.16
Jacke	0.68	2.18	Gras	0.77	1.59
Karton	0.00	1.84	Grundstück	0.76	2.03
Kastanie	0.91	2.14	Haube	-0.10	2.16
Klinke	0.23	1.91	Hocker	0.14	1.95
Liter	0.50	1.89	Imker	1.00	2.17
Locher	-0.14	1.82	Kammer	-0.82	2.05
Malerei	1.00	2.06	Kiefer	0.60	1.84
Masche	-0.85	2.10	Kommode	0.64	1.82
Moos	0.70	1.90	Kreis	0.38	1.89
Motto	0.60	2.19	Linie	0.10	1.84
Pfanne	0.65	1.84	Mensa	-0.05	2.00
Puder	0.65	1.95	Minigolf	0.40	1.78
Punkt	0.18	1.89	Notiz	0.65	2.05
Raum	0.74	2.14	Ofen	1.00	2.18
Reihe	0.00	1.94	Porto	-0.65	1.79
Seife	0.70	1.47	Puppe	0.45	2.00
Sieb	0.36	1.82	Rast	0.90	1.90
Stempel	-0.05	2.00	Rinde	0.35	1.89
Tasse	0.73	1.82	Roggen	0.65	1.79
Taste	0.35	2.13	Schürze	-0.64	2.14
Truhe	0.85	2.14	Sitzung	-0.38	2.11
Vorkommen	0.18	2.05	Suppe	0.60	2.16
Vorstand	-0.94	2.05	Tante	0.80	1.89
Wolle	0.90	2.00	Teil	0.38	1.89
Wort	0.76	2.00	Tomate	0.95	2.18
Zopf	0.77	2.18	Watte	1.00	1.70

Emotional Valence (EMO) and Arousal ratings for target words words as reported in Võ et al. (2009) for the two experimental blocks with distraction.

Tab. S3 – Auditory distractors presented during the retention intervals

Distraction Sound Block 1/2	PLEASURE_ MEAN	AROUSAL_ MEAN	Distraction Sound Block 3/4	PLEASURE_ MEAN	AROUSAL_ MEAN
102	4.63	4.91	373	5.09	4.65
109	6.4	5.64	374	5.6	4.23
113	5.45	4.88	375	5.99	4.48
120	5.2	5.41	377	4.88	4.6
130	4.64	4.93	382	4.33	4.64
132	5.64	4.77	403	5.57	5.56
152	5.23	5.51	410	4.86	5.89
170	5.31	4.6	425	5.09	5.15
225	5.96	4.83	500	4.32	5.4
245	4.18	5.05	700	4.68	4.03
246	4.83	4.65	701	4.95	4.41
251	4.16	5.14	702	4.45	5.37
252	4.01	4.75	705	5.35	4.15
320	4.23	5.48	706	4.16	5.3
322	5.01	4.79	720	4.86	4.18
358	4.52	4.87	722	4.83	4.97
361	5.36	5.01	723	4.52	4.42
364	5.19	5.62	724	5.34	4.91
368	5.15	4.75	728	4.72	4.35
370	5.94	4.44	729	4.3	5.79
374	5.6	4.23	373	5.09	4.65
375	5.99	4.48	374	5.6	4.23

Pleasure and Arousal ratings for auditory distractors as reported in Bradley & Lang (2007) for the two experimental blocks with distraction.

Tab. S4 – Included 1-second segments of EEG-data for first and the second experimental block involving distraction of the control group, the adherent and the non-adherent sample.

Included 1 sec Segments for the first retention interval with distractors					
non-adherent sample (n = 30)		adherent sample (n = 30)		control group (N = 30)	
Begin	End	Begin	End	Begin	End
60	60	60	60	60	60
60	60	38	60	60	60
60	60	60	60	60	60
20	60	60	60	60	60
60	60	60	60	60	60
60	60	60	60	33	60
60	60	60	60	60	60
60	60	60	60	60	60
60	60	60	60	60	60
19	60	60	60	40	60
60	60	60	60	60	60
60	60	60	60	60	60
60	60	60	60	60	60
60	60	60	60	60	60
60	60	60	60	60	60
60	60	60	60	60	60
10	60	60	60	60	60
60	60	60	60	60	60
60	60	60	60	60	60
60	60	60	60	60	60
60	60	60	60	60	60
60	60	60	60	60	60
60	60	60	60	60	60
60	60	60	60	60	60
40	60	60	60	60	60
60	60	60	60	60	60
60	60	60	60	60	60
60	60	60	60	60	60
60	60	60	60	22	60
60	60	60	60	60	60
Included 1 sec Segments the second retention interval with distractors					
non-adherent sample (n = 30)		adherent sample (n = 30)		control group (N = 30)	
Begin	End	Begin	End	Begin	End
60	60	60	60	60	60
60	60	60	60	60	60
60	60	60	60	60	60
60	60	60	60	4	60
60	60	60	60	40	60
60	60	60	60	60	60
60	60	60	60	60	60
40	60	60	60	60	60
60	60	60	60	60	60

60	60	60	60	11	60
60	60	60	60	35	60
60	60	60	60	60	60
60	60	60	60	60	60
60	60	60	60	60	60
60	60	60	60	60	60
60	60	60	60	60	60
60	60	60	60	60	60
40	60	60	60	60	60
60	60	60	60	60	60
60	60	60	60	60	60
60	60	60	60	60	60
60	60	60	60	60	60
60	60	60	60	37	60
60	60	60	60	60	60
60	60	60	60	60	60
60	60	60	60	36	60
60	60	60	60	60	60
60	60	60	60	60	60
60	60	60	60	60	60
60	60	60	60	60	60

In order to avoid biases, EEG-data of face-touches that occurred in the 60 second segment which was to analyze, 10 seconds before to 10 seconds after a facial self-touch were excluded from further EEG-analyses.

Tab. S5 – Mean or quantity and comparisons of demographic as well as experiment-associated data between the non-adherent and the adherent subsample with as well as without distraction

	non-adherent subsample with distraction (N = 11)	adherent subsample with distraction (N = 49)	Teststatistic *Z* (*p*)
Age (in years)	26.55	26.53	$Z = .03$<br>$p = .98$
Sex	F = 7<br>M = 4	F = 23<br>M = 26	$\chi^2 = 1.00$<br>$p = .61$
Group membership	Group 1 = 4<br>Group 2= 7	Group 1 = 26<br>Group 2= 23	$\chi^2 = 1.00$<br>$p = .61$
Daytime of study	Morning = 5<br>Afternoon = 6	Morning = 21<br>Afternoon = 28	$\chi^2 = .02$<br>$p = .99$
	non-adherent subsample without distraction (N = 8)	adherent subsample without distraction (N = 52)	Teststatistic *Z* (*p*)
Age	26.50	26.54	$Z = -.12$<br>$p = .91$
Sex	F = 5<br>M = 3	F = 25<br>M = 27	$\chi^2 = .58$<br>$p = .75$
Group membership	Group 1 = 4<br>Group 2= 4	Group 1 = 26<br>Group 2= 26	$\chi^2 = 0$<br>$p = 1$
Daytime of study	Morning = 6<br>Afternoon = 2	Morning = 20<br>Afternoon = 32	$\chi^2 = 3.77$<br>$p = .15$

F = female, M = male

Tab. S6 – Mean and comparisons between the adherent and the non-adherent sample as well as the control group for questionnaire data

Questionnaire scale	non-adherent sample Mean (SD)	adherent sample Mean (SD)	control group Mean (SD)	Comparison control vs. non-adherent	Comparison control vs. adherent
Neuroticism	20.57 (7.68)	19.00 (6.02)	19.10 (7.52)	$Z = -.84$ $p = .40$	$Z = -.14$ $p = .89$
Extraversion	27.73 (5.26)	27.63 (5.93)	29.33 (6.39)	$Z = 1.27$ $p = .21$	$Z = 1.21$ $p = .23$
Openness	34.23 (7.30)	34.47 (7.23)	35.50 (6.10)	$Z = .75$ $p = .45$	$Z = .10$ $p = .92$
Agreeableness	34.30 (6.19)	35.77 (6.19)	33.83 (5.61)	$Z = -.47$ $p = .64$	$Z = -1.39$ $p = .17$
Conscientiousness	29.67 (6.60)	33.30 (6.76)	30.30 (8.06)	$Z = .50$ $p = .62$	$Z = -1.50$ $p = .13$
ASRS	2.07 (1.31)	1.37 (1.33)	2.00 (1.64)	$Z = -.30$ $p = .76$	$Z = 1.68$ $p = .09$
STAI – State	29.33 (8.21)	29.23 (9.30)	27.03 (8.12)	$Z = -.97$ $p = .33$	$Z = -.89$ $p = .37$
STAI – Trait	35.83 (9.00)	33.00 (9.19)	32.83 (10.85)	$Z = -1.37$ $p = .17$	$Z = -.13$ $p = .89$

Big Five personality traits (Neuroticism, Extraversion, Openness, Agreeableness, Conscientiousness) as measured with the NEO-FFI questionnaire

ASRS = Adult ADHD Self-Report Scale (screening)

STAI = State and Trait Inventory (screening)

Tab. S7 – Statistical results for the EEG-power for the adherent sample

Z (*p*)	EEG Power Comparison – adherent sample<br>Retention Interval 1 with distractors				
Electrode	Delta	Theta	Alpha	Beta	Gamma
Fp1	1.7 (.09)	2.6 (.0068)	-1.29 (.2)	0.48 (.64)	0.3 (.77)
Fp2	1.59 (.11)	2.58 (.0073)	-0.85 (.4)	-0.2 (.85)	0.05 (.96)
F7	0.69 (.5)	0.42 (.68)	-3.65 (.00004)	-0.28 (.79)	0.3 (.77)
F3	0.36 (.73)	0.09 (.93)	-2.93 (.0019)	-1.02 (.32)	-0.36 (.73)
Fz	1.39 (.17)	0.11 (.91)	-3.71 (.00003)	-2.97 (.0016)	-0.01 (.99)
F4	0.38 (.71)	0.38 (.71)	-2.73 (.0043)	-0.5 (.62)	0.03 (.98)
F8	-0.5 (.62)	1.02 (.32)	-2.68 (.005)	-0.32 (.76)	0.42 (.68)
T3	0.44 (.67)	1.8 (.071)	-2.81 (.0031)	2.44 (.012)	2.4 (.014)
C3	0.11 (.91)	0.83 (.41)	-3.32 (.00028)	-1.45 (.15)	0.09 (.93)
Cz	2.48 (.01)	1.02 (.32)	-3.28 (.00035)	-2.66 (.0054)	0.11 (.91)
C4	-0.61 (.55)	-2.01 (.042)	-3.18 (.0006)	-1.26 (.21)	0.3 (.77)
T4	0.77 (.45)	2.17 (.027)	-1.88 (.058)	2.54 (.0085)	2.83 (.0029)
T5	0.71 (.49)	1.18 (.24)	-3.1 (.00089)	0.55 (.59)	2.21 (.024)
P3	1.39 (.17)	1.29 (.2)	-3.22 (.00049)	-2.27 (.02)	1.14 (.26)
Pz	1.82 (.067)	1.2 (.23)	-3.03 (.0012)	-1.16 (.25)	1.43 (.16)
P4	1 (.33)	-0.3 (.77)	-3.51 (.0001)	-1.29 (.2)	1.26 (.21)
T6	0.05 (.96)	-0.5 (.62)	-3.88 (.000008)	0.83 (.41)	3.32 (.00028)
O1	-0.05 (.96)	0.01 (.99)	-3.45 (.00014)	-1.64 (.1)	2.4 (.014)
O2	-0.13 (.9)	-0.22 (.83)	-3.77 (.00002)	-1.43 (.16)	2.58 (.0073)
Z (*p*)	EEG Power Comparison – adherent sample<br>Retention Interval 2 with distractors				
Electrode	Delta	Theta	Alpha	Beta	Gamma
Fp1	2.4 (.014)	3.1 (.00089)	-1.41 (.16)	1.68 (.094)	1.24 (.22)
Fp2	2.21 (.024)	2.95 (.0017)	-0.71 (.49)	1.33 (.19)	1.06 (.3)
F7	0.85 (.4)	0.77 (.45)	-2.77 (.0037)	-1 (.33)	-0.13 (.9)
F3	0.79 (.44)	0.98 (.34)	-3.32 (.00028)	-0.26 (.8)	0.69 (.5)
Fz	1.06 (.3)	0.22 (.83)	-3.49 (.00011)	-2.42 (.013)	0.77 (.45)
F4	0.63 (.54)	0.98 (.34)	-3.03 (.0012)	0.09 (.93)	1 (.33)
F8	-0.34 (.74)	1.06 (.3)	-2.31 (.018)	0.52 (.61)	0.55 (.59)
T3	1 (.33)	0.52 (.61)	-2.73 (.0043)	1.9 (.055)	1.92 (.052)
C3	1.26 (.21)	0.44 (.67)	-4.21 (.0000004)	-2.77 (.0037)	-0.71 (.49)
Cz	2.38 (.015)	1.53 (.13)	-3.28 (.00035)	-2.87 (.0024)	-0.81 (.43)
C4	0.48 (.64)	-1.2 (.23)	-4.35 (.00000008)	-2.58 (.0073)	0.17 (.86)
T4	0.48 (.64)	0.42 (.68)	-3.22 (.00049)	2.03 (.04)	2.25 (.021)
T5	1.74 (.082)	0.75 (.46)	-3.88 (.000008)	-0.61 (.55)	1.59 (.11)
P3	2.25 (.021)	1.74 (.082)	-3.49 (.00011)	-2.01 (.042)	0.17 (.86)
Pz	1.88 (.058)	1.66 (.098)	-2.56 (.0079)	-1.82 (.067)	0.36 (.73)
P4	1.45 (.15)	1.04 (.31)	-2.77 (.0037)	-0.75 (.46)	1.45 (.15)
T6	1.31 (.2)	0.34 (.74)	-2.17 (.027)	1.51 (.13)	2.01 (.042)
O1	1.41 (.16)	0.42 (.68)	-2.97 (.0016)	0.69 (.5)	2.48 (.01)
O2	1.33 (.19)	0.77 (.45)	-1.78 (.074)	1.33 (.19)	2.38 (.015)

Results of the Wilcoxon signed-rank test

adherent sample = participants, who did not touch their face during the third or fourth experimental run, i.e. after being instructed to refrain from face-touching.

Tab. S8 – Statistical results for the EEG-power for the non-adherent sample

Z (*p*)	EEG Power Comparison – non-adherent sample Retention Interval 1 with distractors				
Electrode	Delta	Theta	Alpha	Beta	Gamma
Fp1	1.24 (.22)	1.8 (.071)	-0.94 (.36)	0.24 (.82)	0.48 (.64)
Fp2	0.24 (.82)	1.24 (.22)	-0.34 (.74)	0.92 (.37)	-0.98 (.34)
F7	1.49 (.14)	1.39 (.17)	-2.38 (.015)	-1.78 (.074)	-1.7 (.09)
F3	1.37 (.18)	0.79 (.44)	-2.42 (.013)	-1.96 (.047)	-2.07 (.036)
Fz	1 (.33)	0.32 (.76)	-2.33 (.017)	-2.29 (.019)	-1.78 (.074)
F4	-0.4 (.7)	-0.09 (.93)	-2.68 (.005)	-2.05 (.038)	-2.23 (.023)
F8	1.39 (.17)	1.94 (.05)	-2.73 (.0043)	-2.15 (.029)	-1.55 (.12)
T3	1 (.33)	1.86 (.061)	-3.32 (.00028)	1 (.33)	0.98 (.34)
C3	0.85 (.4)	1.7 (.09)	-2.46 (.011)	-1.7 (.09)	-1.9 (.055)
Cz	1.43 (.16)	1.08 (.29)	-1.82 (.067)	-2.13 (.03)	-2.46 (.011)
C4	1.29 (.2)	0.89 (.38)	-2.29 (.019)	-1 (.33)	-1.64 (.1)
T4	0.48 (.64)	0.09 (.93)	-1.92 (.052)	1 (.33)	1.66 (.098)
T5	0.55 (.59)	0.28 (.79)	-3.07 (.00098)	-1 (.33)	0.89 (.38)
P3	0.57 (.58)	0.48 (.64)	-2.64 (.0059)	-1.55 (.12)	-0.61 (.55)
Pz	1.29 (.2)	0.81 (.43)	-2.95 (.0017)	-2.42 (.013)	-0.98 (.34)
P4	0.98 (.34)	0.42 (.68)	-2.75 (.004)	-0.75 (.46)	-1.18 (.24)
T6	1.37 (.18)	0.13 (.9)	-2.01 (.042)	0.59 (.57)	1.2 (.23)
O1	0.13 (.9)	-0.03 (.98)	-2.93 (.0019)	-1.29 (.2)	0.55 (.59)
O2	-0.07 (.94)	-0.22 (.83)	-3.07 (.00098)	-0.63 (.54)	0.63 (.54)
Z (*p*)	EEG Power Comparison – non-adherent sample Retention Interval 2 with distractors				
Electrode	Delta	Theta	Alpha	Beta	Gamma
Fp1	2.56 (.0079)	1.98 (.045)	-1.94 (.05)	-0.2 (.85)	0.46 (.65)
Fp2	1.82 (.067)	1.9 (.055)	-1.04 (.31)	-0.5 (.62)	-0.03 (.98)
F7	1.14 (.26)	0.22 (.83)	-3.79 (.00001)	-1.9 (.055)	-1.1 (.28)
F3	1.45 (.15)	2.11 (.032)	-3.38 (.0002)	-2.29 (.019)	-1 (.33)
Fz	0.48 (.64)	0.65 (.53)	-3.55 (.00008)	-2.38 (.015)	0.2 (.85)
F4	1.33 (.19)	-0.24 (.82)	-3.38 (.0002)	-1.43 (.16)	0.09 (.93)
F8	1.96 (.047)	1.2 (.23)	-3.63 (.00005)	-2.44 (.012)	-1.16 (.25)
T3	-0.46 (.65)	-0.67 (.51)	-2.75 (.004)	1.98 (.045)	2.68 (.005)
C3	1.9 (.055)	1.9 (.055)	-3.69 (.00003)	-3.18 (.0006)	0.01 (.99)
Cz	0.96 (.35)	1.08 (.29)	-3.53 (.00009)	-3.36 (.00023)	-2.52 (.0091)
C4	1 (.33)	-0.73 (.47)	-3.96 (.000004)	-3.36 (.00023)	-1.02 (.32)
T4	-0.11 (.91)	0.65 (.53)	-3.12 (.00081)	1.72 (.085)	2.54 (.0085)
T5	1.16 (.25)	-0.11 (.91)	-3.18 (.0006)	0.73 (.47)	2.44 (.012)
P3	1.12 (.27)	0.67 (.51)	-3.84 (.00001)	-3.22 (.00049)	0.61 (.55)
Pz	0.96 (.35)	0.48 (.64)	-4.08 (.000001)	-2.7 (.0047)	0.59 (.57)
P4	0.83 (.41)	0.73 (.47)	-3.73 (.00002)	-2.89 (.0022)	0.3 (.77)
T6	0.81 (.43)	-0.32 (.76)	-2.56 (.0079)	0.92 (.37)	2.07 (.036)
O1	-0.05 (.96)	1.08 (.29)	-1.68 (.094)	0.63 (.54)	1.84 (.064)
O2	0.46 (.65)	0.81 (.43)	-2.4 (.014)	0.34 (.74)	1.37 (.18)

Results of the Wilcoxon signed-rank test

non-adherent sample = participants, who touched their face during the third or fourth experimental run, i.e. after being instructed to refrain from face-touching.

Tab. S9 – Statistical results for the EEG-power for the control group

Z (*p*)	EEG Power Comparison – control group<br>Retention Interval 1 with distractors				
Electrode	Delta	Theta	Alpha	Beta	Gamma
Fp1	2.25 (.021)	1.76 (.078)	-1.9 (.055)	-0.87 (.39)	-0.52 (.61)
Fp2	2.4 (.014)	1.74 (.082)	-1.31 (.2)	0.61 (.55)	-0.07 (.94)
F7	1.53 (.13)	0.63 (.54)	-2.58 (.0073)	-0.17 (.86)	0.61 (.55)
F3	1.7 (.09)	0.5 (.62)	-3.73 (.00002)	-2.68 (.005)	0.26 (.8)
Fz	0.98 (.34)	0.94 (.36)	-4.17 (.0000006)	-3.71 (.00003)	-0.81 (.43)
F4	1.82 (.067)	0.22 (.83)	-3.82 (.00001)	-2.17 (.027)	0.15 (.88)
F8	1.82 (.067)	1.02 (.32)	-2.23 (.023)	0.13 (.9)	0.94 (.36)
T3	2.15 (.029)	0.71 (.49)	-2.23 (.023)	2.27 (.02)	2.77 (.0037)
C3	2.05 (.038)	0.28 (.79)	-3.73 (.00002)	-2.03 (.04)	1 (.33)
Cz	1.72 (.085)	-0.11 (.91)	-3.36 (.00023)	-3.2 (.00054)	-1.22 (.23)
C4	1.41 (.16)	-1.49 (.14)	-4.08 (.000001)	-2.91 (.002)	0.89 (.38)
T4	1.22 (.23)	0.96 (.35)	-2.15 (.029)	2.07 (.036)	2.23 (.023)
T5	2.83 (.0029)	0.61 (.55)	-4.23 (.0000003)	-0.05 (.96)	3.26 (.00039)
P3	2.11 (.032)	0.63 (.54)	-4.06 (.000002)	-3.18 (.0006)	1.92 (.052)
Pz	2.15 (.029)	0.71 (.49)	-4.29 (.0000002)	-3.26 (.00039)	1.1 (.28)
P4	2.36 (.016)	0.4 (.7)	-4.43 (.00000003)	-2.99 (.0014)	1.51 (.13)
T6	1.8 (.071)	0.59 (.57)	-3.9 (.000007)	0.46 (.65)	3.28 (.00035)
O1	1.78 (.074)	0.32 (.76)	-3.96 (.000004)	-1.57 (.12)	1.88 (.058)
O2	1.04 (.31)	-0.48 (.64)	-3.57 (.00007)	-0.75 (.46)	2.85 (.0026)
Z (*p*)	EEG Power Comparison – control group<br>Retention Interval 2 with distractors				
Electrode	Delta	Theta	Alpha	Beta	Gamma
Fp1	2.46 (.011)	3.69 (.00003)	0.57 (.58)	0.03 (.98)	-0.09 (.93)
Fp2	2.73 (.0043)	3.05 (.0011)	0.92 (.37)	0.28 (.79)	0.26 (.8)
F7	1.35 (.18)	1.33 (.19)	-0.38 (.71)	0.15 (.88)	-0.57 (.58)
F3	1.16 (.25)	0.5 (.62)	-2.23 (.023)	-0.61 (.55)	-0.42 (.68)
Fz	0.4 (.7)	1.06 (.3)	-2.19 (.026)	-0.83 (.41)	0.69 (.5)
F4	1.7 (.09)	0.2 (.85)	-1.88 (.058)	0.55 (.59)	0.65 (.53)
F8	1.64 (.1)	1.8 (.071)	-0.36 (.73)	1.24 (.22)	1.35 (.18)
T3	1.45 (.15)	0.81 (.43)	-0.32 (.76)	3.03 (.0012)	3.03 (.0012)
C3	2.42 (.013)	1.18 (.24)	-3.45 (.00014)	0.22 (.83)	0.77 (.45)
Cz	1.37 (.18)	0.65 (.53)	-2.75 (.004)	-0.75 (.46)	0.59 (.57)
C4	0.89 (.38)	-0.59 (.57)	-3.24 (.00044)	-0.59 (.57)	0.5 (.62)
T4	-0.57 (.58)	0.26 (.8)	-2.54 (.0085)	2.46 (.011)	2.31 (.018)
T5	1.2 (.23)	0.22 (.83)	-2.29 (.019)	1.64 (.1)	2.5 (.0097)
P3	2.83 (.0029)	1.45 (.15)	-1.2 (.23)	-0.38 (.71)	0.69 (.5)
Pz	2.17 (.027)	0.96 (.35)	-2.11 (.032)	-1.06 (.3)	0.92 (.37)
P4	2.4 (.014)	0.79 (.44)	-0.92 (.37)	-0.15 (.88)	1.39 (.17)
T6	1.66 (.098)	0.5 (.62)	-2.46 (.011)	2.54 (.0085)	2.23 (.023)
O1	1.22 (.23)	0.57 (.58)	-0.89 (.38)	0.77 (.45)	2.36 (.016)
O2	1.12 (.27)	0.59 (.57)	-1 (.33)	1.74 (.082)	2.95 (.0017)

Results of the Wilcoxon signed-rank test
control group = participants, who did not receive any instruction concerning face-touch behavior.
